# Crystal Structure of a Group I Energy Coupling Factor Vitamin Transporter S Component in Complex with Its Cognate Substrate

**DOI:** 10.1016/j.chembiol.2016.06.008

**Published:** 2016-07-21

**Authors:** Inokentijs Josts, Yasser Almeida Hernandez, Antonina Andreeva, Henning Tidow

**Affiliations:** 1 The Hamburg Centre for Ultrafast Imaging (CUI), Institute for Biochemistry and Molecular Biology, University of Hamburg, Martin-Luther-King-Platz 6, 20146 Hamburg, Germany; 2 Department of Chemistry, Institute for Biochemistry and Molecular Biology, University of Hamburg, Martin-Luther-King-Platz 6, 20146 Hamburg, Germany; 3 MRC Laboratory of Molecular Biology, Francis Crick Avenue, Cambridge CB2 0QH, UK

## Abstract

Energy coupling factor (ECF) transporters are responsible for the uptake of essential scarce nutrients in prokaryotes. This ATP-binding cassette transporter family comprises two subgroups that share a common architecture forming a tripartite membrane protein complex consisting of a translocation component and ATP hydrolyzing module and a substrate-capture (S) component. Here, we present the crystal structure of YkoE from *Bacillus subtilis*, the S component of the previously uncharacterized group I ECF transporter YkoEDC. Structural and biochemical analyses revealed the constituent residues of the thiamine-binding pocket as well as an unexpected mode of vitamin recognition. In addition, our experimental and bioinformatics data demonstrate major differences between YkoE and group II ECF transporters and indicate how group I vitamin transporter S components have diverged from other group I and group II ECF transporters.

## Highlights

• The crystal structure of a group I ECF transporter S component was determined
• The thiamine-bound structure was determined in lipidic environment
• The structure reveals major differences to group II S components
• CGMD simulations indicate the orientation of the protein in the membrane

## Graphical Abstract

**Figure:**
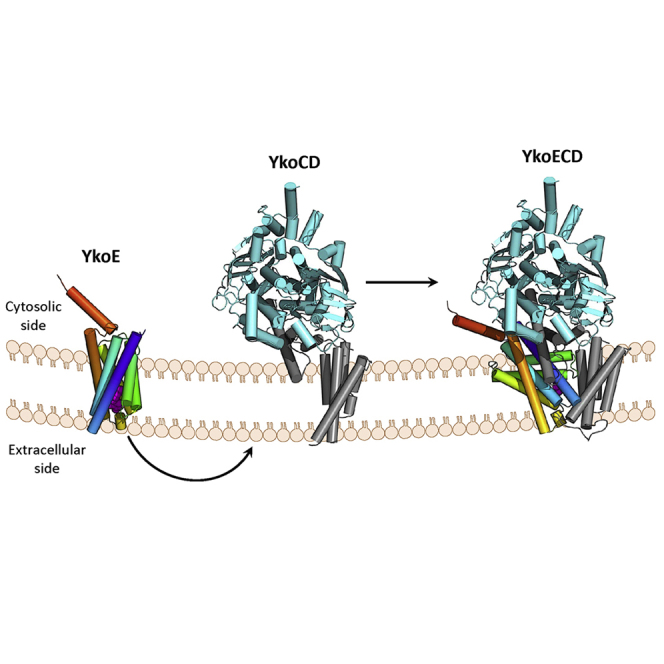

## Teaser

Josts et al. determined the crystal structure of a substrate-bound group I ECF vitamin transporter S component, which revealed major differences to group II S components and indicates how group I S components have diverged from other group I and II ECF transporters.

## Introduction

Energy coupling factor (ECF) transporters form a large superfamily of prokaryotic membrane translocation systems involved in the uptake of scarce nutrients and trace elements from the environment. They form a modular complex consisting of two integral transmembrane proteins, a T component, and an S component that form the conduit for the substrate, coupled to a soluble ATP-binding cassette (ABC) protein (Rodionov et al., 2009). The S component is involved in substrate recognition and generally interacts with its substrate molecule with very high affinity (Duurkens et al., 2007, Erkens and Slotboom, 2010). The ABC component has two nucleotide-binding domains (NBD) and drives the substrate translocation by utilizing ATP hydrolysis; the NBD proteins are coupled with the T component, which acts as a scaffold and can interact with the corresponding S component of the ECF modules and confer conformational rearrangement within the complex, coordinating ATP hydrolysis and substrate translocation (Zhang et al., 2014, Xu et al., 2013, Swier et al., 2016).

**Figure 1:**
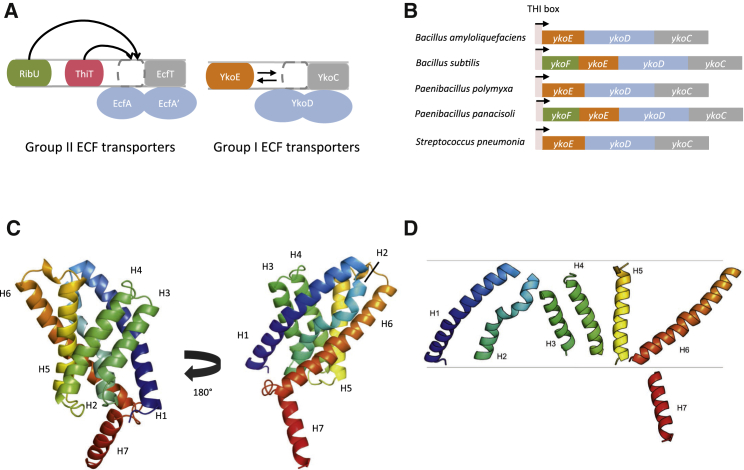
Overall Structure of YkoE (A) Comparison of the architectures between group I and group II ECF transporters. Group II ECF modules can share several different S components, whereas group I S components are specific for their cognate ECF module, with all partners generally found in an operon together. (B) Genetic organization of *ykoEDC* operon. The entire operon is under the regulation of the THI box, a thiamine-responsive riboswitch. The function of YkoF (green), an oligomeric, soluble thiamine-binding protein is currently unclear. (C) Ribbon depiction of the overall conformation of YkoE. (D) Cartoon representation of the orientation of individual helices in YkoE.

ECF modules can be classified into two distinct groups (Figure 1A). The best characterized are the group II ECF transporters, which share a common T component (EcfT) and NBD proteins (EcfA and A′) with different S components (EcfS), such as ThiT, RibU, BioY, and PanT, forming distinct interacting partners (Zhang et al., 2010, Erkens et al., 2011, Berntsson et al., 2012, Karpowich et al., 2015). Despite high structural similarity, group II S components share very low sequence similarity, with the most conserved motif being the residues interacting with the T component. The less characterized group I ECF transporters form a dedicated tripartite membrane protein complex wherein all the constitutive components are generally encoded together in one operon. The basic molecular organization of group I ECF transporters is similar to the group II ECF transporters. Moreover, mechanistic crosslinking and spectroscopic studies exist for the group I BioMNY complex (Neubauer et al., 2011, Finkenwirth et al., 2013, Finkenwirth et al., 2015). However, only one high-resolution structure of a group I S component is available to date. NikM, the S component of an ECF type nickel/cobalt transporter, contains an additional N-terminal transmembrane helix that is crucial to the coordination of the nickel or cobalt ion (Yu et al., 2014). It is currently unclear whether all group I S components contain common structural features and high-resolution structural details of their interaction with the T components are missing.

The *yko* operon encodes for a group I ECF module, where YkoE acts as the substrate-capture S component, YkoC as the T component and YkoD represents the ABC component consisting of two fused ATPase domains (Figure 1A). The *ykoEDC* operon is found in Gram-positive bacteria and is under control of a THI box riboswitch, implicating the complex in the transport of thiamine or a thiamine precursor across the bacterial membrane (Figure 1B). Little is known about the substrate specificity of the YkoEDC complex, however some studies have shown that the complex is capable of transporting thiamine (Schyns et al., 2005). Moreover, some bacterial species possess an additional gene within the operon, a soluble thiamine-binding protein YkoF (Devedjiev et al., 2004).

Despite extensive research into ECF modules, the mechanistic details of substrate transport remain highly elusive. The structures of the entire ECF modules for folate and pantothenate transporters reveal an almost parallel orientation of the S component to the membrane, indicating a toppling mechanism during substrate translocation (Xu et al., 2013, Swier et al., 2016, Zhang et al., 2014). As these structures were free of nucleotide and substrate, they most likely represent a state after ATP hydrolysis and substrate release (Zhang, 2013, Slotboom, 2014, Finkenwirth et al., 2015). In the course of the transport cycle, the substrate-bound S component must associate with EcfT and NBD proteins and is assumed to topple over.

Here, we present the first crystal structure of a group I ECF vitamin transporter S component, YkoE. While the overall conformation of YkoE resembles those of group II ECF transporters, this group I S component contains several unique structural features. We combine structural analysis with bioinformatics and molecular dynamic simulations in order to probe the impact of these additional structural features on the YkoEDC complex formation and mechanism of vitamin transport.

## Results

### Overall Structure

**Figure 2:**
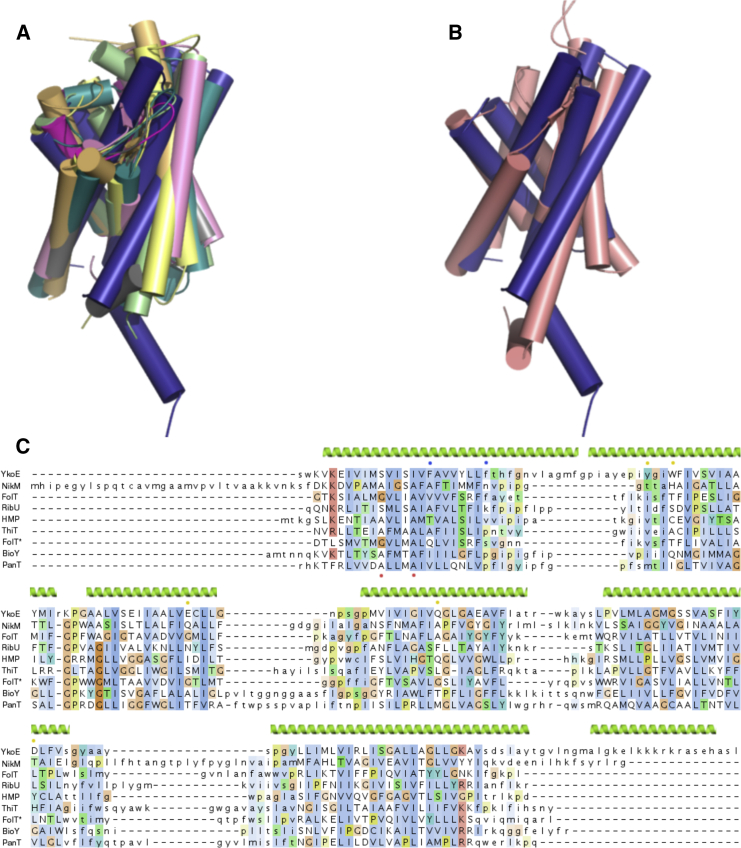
Structure Superposition of S Components (A) Superposition of the structure of YkoE (dark blue) and the structures of group II S components colored as follows: FolT with bound folate (PDB: 4Z7F) in pink, RibU (PDB: 3P5N) in light green, HMP (PDB: 4HZU) in magenta, ThiT (PDB: 3RLB) in yellow, apoFolT (PDB: 4HUQ) in gray, BioY (PDB: 4DVE) in green, PanT (PDB: 4RFS) in orange. (B) Superposition of YkoE (dark blue) and NikM (light pink). (C) Structure-based multiple sequence alignment of group I and II S components. The secondary structural elements of YkoE are shown on the top. Structurally equivalent residues are shown in uppercase. The residues contributing to the conserved ΦXXXA motif are indicated with red dots at the bottom of the alignment, the YkoE residues that interact with the thiamine are indicated with yellow dots, and the Phe residues that obscure the groove with blue dots.

**Figure 3:**
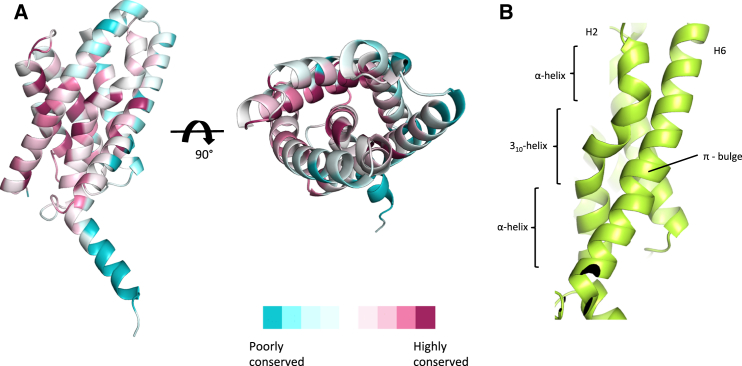
Structural Overview of the Conserved Motifs in the Group I S Component YkoE (A) Substrate cavity and interhelical contacts are the most conserved regions of YkoE. Conservation of amino acid residues was analyzed using ConSurf (Ashkenazy et al., 2010). 980 non-redundant sequences of YkoE homologs were used in the alignment to emphasize the most conserved regions of the structure. Highly conserved residues are depicted as burgundy patches; moderately conserved side chains are shown in light pink. Weakly conserved residues are colored in cyan. Residues that exhibit some degree of conservation among the 980 homologs of YkoE are in white. (B) Ribbon representation of the packing conformations of helix H2 and helix H6. Helix H2 is comprised of α-3_10_-α helical elements that allow it to pack tightly against helix H6, thereby closing the cavity from the cytoplasmic side. The highly conserved π bulge is in the middle of helix H6.

To gain insights into the function of the YkoEDC ECF transporter, we solved the crystal structure of its S component YkoE. The gene was cloned from several bacterial species, and the protein was expressed and purified to homogeneity. YkoE failed to crystallize using the traditional vapor-diffusion methods after screening several different homologs. However, YkoE from *Bacillus subtilis* could be readily crystallized using the lipidic cubic phase (LCP) method. The structure was solved using single-wavelength anomalous dispersion (SAD) with selenomethionine-labeled YkoE to 1.95 Å resolution. The electron density from native crystals was of sufficient quality to build the entire molecule of YkoE with the exception of the four N-terminal amino acids (Figure S1A). The structure of YkoE revealed a six helical transmembrane domain with the overall fold reminiscent of S components from group II ECF transporters (root-mean-square deviation between YkoE and other S components ranges between 2.6 and 3.6 Å) (Figures 1C and 2A). YkoE possesses an additional C-terminal helix that presumably protrudes toward the cytosol and lies perpendicular to the lipid bilayer (Figure 1D). The present orientation of the helix is likely stabilized by the crystallographic contacts between neighboring molecules (Figure S1B). The six hydrophobic helices form a tight fold with an open cavity with a volume of 545 Å^3^ facing the extracellular part of the membrane. The most conserved amino acid residues in YkoE map to the interior of the cavity as well as residues involved in the interhelical packing within the molecule (Figure 3A). In YkoE, helix H1 is highly extended with a bend in the middle, leading into a sharp turn joining to helix H2 (Figure 3B). Helix H2 possesses a conserved Pro44 that breaks up the α-helical backbone, giving rise to a kink in the helix that leads into a 3_10_ helical conformation, returning to a regular α-helical backbone after a short amino acid stretch (Figures 1D and 3B). Such a structural feature is reminiscent of helix H4 in ThiT where the π bulge dictates the conformation of the residues forming the thiamine-binding site (Erkens et al., 2011). In YkoE, helix H2 packs very tightly against helix H6, which bears a highly conserved π bulge that introduces an additional kink at the nearly invariant Gly47 residue in helix H2, and thus reversing the 3_10_ helical stretch to an α-helical one (Figure 3B). This packing arrangement, together with the surrounding helices H3, H4, and H5, creates a funnel-like substrate-binding cavity.

### Thiamine Coordination

**Figure 4:**
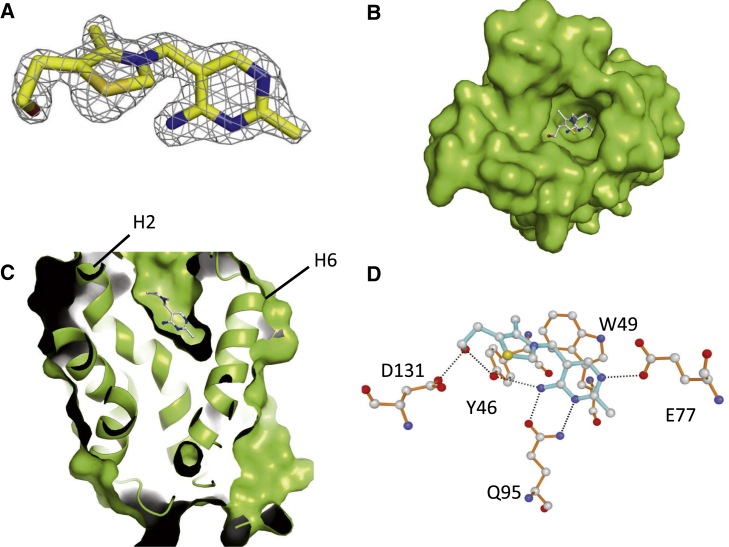
Thiamine-Binding Site of YkoE (A) Electron density of thiamine from the 2F_o_ − F_c_ map contoured at 1.5 σ. (B) Top view of the surface of YkoE with thiamine bound in the substrate cavity. (C) Cross-section of YkoE with thiamine localized within the binding pocket. Black lines indicate the positions of helix H2 and helix H6. (D) Coordination of thiamine (cyan) by residues within the substrate-binding pocket (orange). Hydrogen bonds are shown as black dashes. Carbon atoms are colored gray, nitrogen atoms are purple, oxygen atoms are red, and the sulfur atom is light orange.

**Figure 5:**
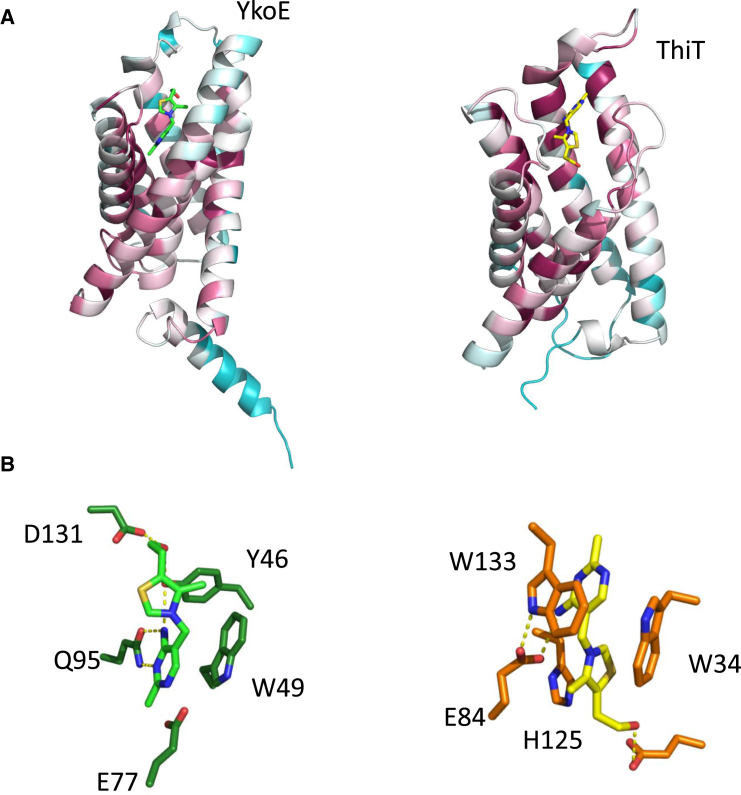
Side by Side Comparison of YkoE and ThiT Both proteins are colored by conservation. (A) YkoE (left) and ThiT (right) with bound thiamine. (B) The constituent residues of the YkoE (left) and ThiT (right) thiamine binding pocket that interact with thiamine.

During the initial stages of refinement, the density for thiamine became apparent and allowed the modeling of the full molecule unambiguously (Figure 4A). The thiamine molecule is present at the base of the cavity found in the extracellular part of the membrane (Figures 4B and 4C). The pyrimidine group forms π-stacking interactions with a highly conserved Trp49 located at the kink of helix H2. In addition, the pyrimidine group is coordinated by H bonds by highly conserved Glu77 and Gln95 residues located on helix H3 and H4, respectively. The thiazole ring of thiamine forms H-bonding interactions with Asp131 and Tyr46 (Figure 4D). The residues coordinating the pyrimidine moiety of thiamine are more conserved than those coordinating the thiazole moiety (Figure S2A). The orientation of the thiamine in the YkoE binding pocket differs significantly from that of the thiamine bound to ThiT, a group II ECF S component (Erkens et al., 2011). The thiazole moiety of thiamine in ThiT points to the bottom of the binding pocket and the pyrimidine moiety faces the extracellular side (Figures 5A and S3). In contrast, the thiamine bound to YkoE is in a reverse orientation and located much deeper in the binding pocket. There are also differences between the key interactions for thiamine binding in the YkoE and ThiT binding pockets. In ThiT, the thiazole ring is sandwiched between the conserved Trp34 and His125 located on loop L1 and helix H5, respectively. In addition, the Glu84 residue in helix H4 forms a hydrogen bond with the pyrimidine moiety and Trp133 located at the cap of helix H5 makes a stacking interaction (Figure 5B). The latter is reminiscent of the interaction between the conserved Trp49 and the pyrimidine ring of the thiamine in the YkoE structure. The conformation of the thiamine molecule in the binding sites of YkoE and ThiT is almost identical, both molecules having the low-energy F conformation as defined by the dihedral angles ϕ_T_ (C5′-C3,5′-N3-C2) and ϕ_P_ (N3-C3,5′-C5′-C4′) (Pletcher et al., 1977). The thiamine-binding crevice in YkoE is open and not protected by lid closure mediated by loop L1 as observed for several group II ECF S components (Figures 4B and S3) (Zhang et al., 2010, Erkens et al., 2011, Zhao et al., 2015).

### Biochemical Characterization of Thiamine Binding to YkoE

**Figure 6:**
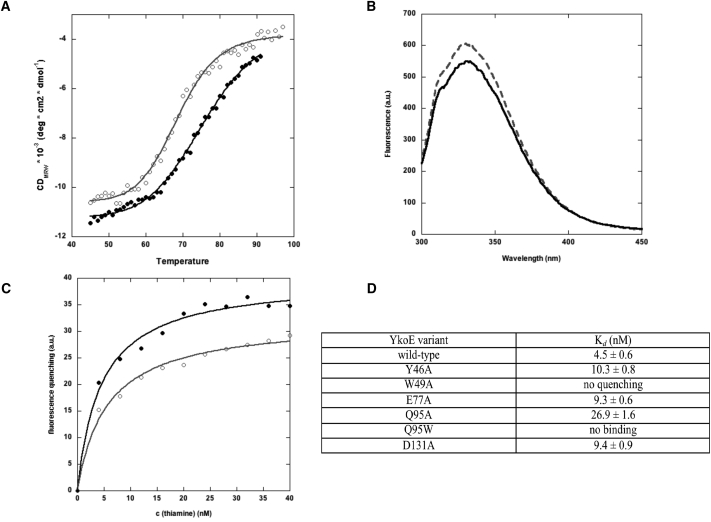
Binding of Thiamine to YkoE (A) Temperature-induced unfolding of YkoE_M9_ (open circles) and YkoE_TB_ (filled circles) monitored by CD spectroscopy. CD signal at 222 nm was recorded in 30 mM Tris, 200 mM NaCl (pH 7.1). The continuous line in each plot corresponds to a standard two-state unfolding model. (B) Fluorescence spectra of 200 nM YkoE_M9_ in the absence (dashed line) and presence of a saturating amount of thiamine (solid line; 800 nM). (C) Titration of 100 nM YkoE_M9_ with thiamine. Intrinsic protein fluorescence was measured with excitation wavelength of 280 nm and emission wavelength of 340 nm (filled circles) and 350 nm (open circles), respectively. The continuous line in each plot corresponds to a single-site binding model fit. (D) Table summarizing the binding affinities for various YkoE variants.

*Escherichia coli* is able to synthesize thiamine in its cytoplasm, therefore we decided to investigate whether YkoE co-purifies with its substrate in the pre-bound form as reported for several other S components (Erkens and Slotboom, 2010, Berntsson et al., 2012). We expressed the protein in standard terrific broth as well as M9 minimal media without the addition of thiamine as a co-factor. In both instances, thiamine could be detected using MALDI-TOF mass spectrometry from the denatured YkoE protein, confirming that, like for other S components, the affinity between YkoE and thiamine is very tight. To investigate whether there was a difference in the populations between the pre-bound versus apo-YkoE, we performed temperature melting circular dichroism (CD) experiments to assess the stability of proteins overexpressed under different conditions. YkoE_TB_ gave a T_m_ of 73°C whereas YkoE_M9_ had a T_m_ value of 68°C, which suggested that YkoE produced in M9 minimal media contained a substantial population of apo-YkoE molecules (Figure 6A). To confirm that the difference in T_m_ between the proteins is due to the presence of pre-bound thiamine, we added an excess of thiamine to YkoE_M9_ and repeated the CD melting experiments. The measured T_m_ of YkoE_M9_ supplemented with excess thiamine was 75°C, which confirmed that, when overexpressed in M9 minimal media, a substantial proportion of YkoE is in its apo form. We then proceeded to investigate the thiamine-YkoE_M9_ interactions using intrinsic Trp fluorescence measurements. The addition of excess thiamine led to the quenching of Trp fluorescence in YkoE and allowed us to determine an approximate dissociation constant (K_*d*_) of 4.5 nM for YkoE_M9_-thiamine complex formation (Figures 6B and 6C). The YkoE_W49A_ mutant did not exhibit any Trp quenching in response to the thiamine titration. Substitution of other thiamine coordinating residues with alanine (namely YkoE_E77A_, YkoE_D131A_, YkoE_Q95A_, YkoE_Y46A_) resulted in 2- to 5-fold weaker binding with the Q95A mutation showing the largest effect on affinity (Figure 6D). In addition, introduction of a bulky Trp side chain (YkoE_Q95W_ mutant) abolished thiamine binding completely, possibly by causing a steric clash with the pyrimidine group at the bottom of the binding pocket. Altogether, the mutagenesis studies presented here corroborate with the observed orientation of thiamine in the substrate-binding site of YkoE *in crystallo*.

### Orientation of YkoE in the Membrane

The orientation of ECF transporter S components in the membrane is highly debated. So far, all individual group II S components have been crystallized in detergent conditions and the orientation of monomers with respect to each other in the asymmetric unit is usually incompatible with the formation of a continuous lipid bilayer (Erkens et al., 2011). YkoE is the first S component that was crystallized in LCP. The crystal packing shows extensive head-to-tail interactions forming type I membrane protein crystals (Figure S1B) and suggests a standard orientation in the membrane, i.e., with helices perpendicular to the membrane plane. Two lipid molecules could be assigned in the electron density of YkoE aligning with the transmembrane helices (Figure S1C), thus further supporting this orientation of YkoE in the membrane bilayer. In contrast, in available crystal structures of entire ECF complexes, the S components are positioned in an almost horizontal orientation in the bilayer, perpendicular to the T components (Xu et al., 2013, Zhang et al., 2014), suggesting a toppling mechanism as the basis for the import of substrates during their catalytic cycle (Slotboom, 2014).

In order to investigate the basis for the toppling mechanism, we performed coarse-grained molecular dynamics (CGMD) simulations of YkoE in lipid bilayers. Using a vertical (perpendicular) starting orientation (standard), YkoE is stable during 2 μs simulations (Figure S4A). In contrast, YkoE positioned in a horizontal (parallel) orientation in the lipid bilayer, an orientation reminiscent of S components in intact ECF complexes, rapidly turns by 60° to adopt a stable perpendicular/vertical orientation (Figure S4B). We further investigated the role of the highly charged C-terminal helix of YkoE. In the absence of the C-terminal helix, YkoE also turns into a stable perpendicular/vertical orientation, although with a significant delay compared to wild-type YkoE, suggesting that electrostatic forces between the C-terminal helix H7 of YkoE and the phosphate head groups of the lipid bilayer may accelerate reorientation of the protein in the bilayer (Figure S4C).

To investigate whether these results are a special feature of YkoE or also occur in other group I or group II S components, we performed similar CGMD simulations with all ECF S components with known structure (Figure S5). All investigated S components return to their standard orientation within 1 μs simulation. While most S components begin to turn immediately, YkoE-ΔC, a truncated version of NikM, and RibU show a significant delay. These CGMD simulations also suggest that isolated S components are unlikely to topple over by themselves, but rather require T-component association for toppling. As all investigated S components are positively charged in their intracellular side (Figure S6), in accordance with the positive-inside rule (Heijne, 1986), it is tempting to speculate that a positively charged cytoplasmic/intracellular region is responsible for the rapid reorientation of S components in the membrane (after substrate release and dissociation from the T component). A partition between positive inside and a more uncharged/hydrophobic outside is a general feature of ECF S components and may facilitate the integration of extracellular regions in the bilayer during the proposed toppling mechanism by lowering the energetic barrier.

## Discussion

The structure of YkoE provides the first insight into the S component of a group I vitamin transporter and how it relates to other group I and II ECF S components. Despite having a common evolutionary origin, evidenced by their global structural similarity and binding site architecture, group I and group II S components differ in several aspects. Since distinct group II S components use the same ECF module, they have evolved to compete for the common T component depending on their substrate load. This probably exerts a strong evolutionary pressure to maintain structural complementarity of the interface between ECF S and T components, and therefore group II S components share certain sequence and structural features. In contrast, group I S components associate with their own distinct ECF module, the components of which can co-evolve, acquire new features, and diverge independently from other group I and group II transporters. The latter statement is supported by the comparison of the NikM and YkoE structures that reveal lack of conservation, and differences in the length of their loops, interhelical packing, and additional N- and C-terminal helices (Figure 2B). Analysis of the structures of group II ECF transporters suggests that the main interactions between S and T components involve helix H1, the groove between helices H1 and H6, the loop connecting helices H5 and H6 from the S component, and coupling helices CH2, CH3, and transmembrane helices from the T component (Xu et al., 2013, Zhang et al., 2014, Swier et al., 2016). Structure superposition of the T components suggests small conformational changes at the transmembrane helices that probably allow a certain degree of freedom in order to accommodate distinct S components. All group II S components possess a ΦxxxA motif (where Φ is a small residue) in their helix H1 that interacts with helix CH2 of the T component. The conservation of this motif is not strict and individual mutations are tolerated in several group I and group II S components (Zhang et al., 2014, Erkens et al., 2011, Finkenwirth et al., 2015). Another characteristic feature of the interface between group II S and T components is a deep groove defined by helices H1 and H6 of the S component, which serves as a platform on which helix CH3 of the T component docks via two highly conserved Phe residues (Figure S7A). Helices CH2 and CH3 are the most conserved parts of the group II T components, underpinning their importance for protein function.

**Figure 7:**
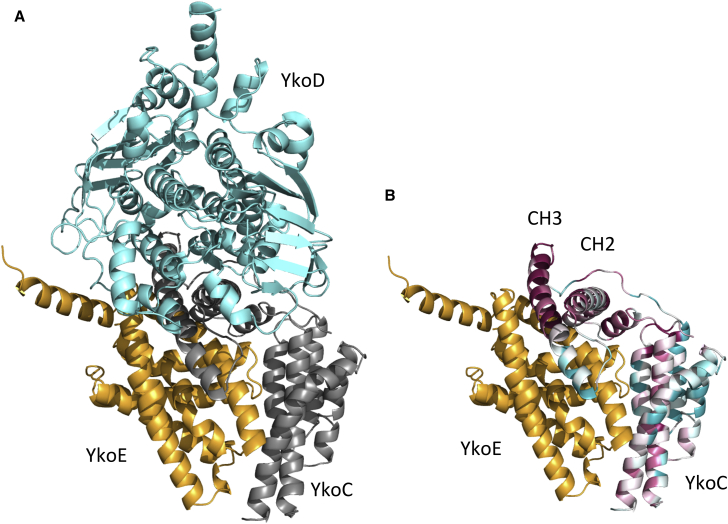
Modeling of YkoEDC Complex (A) Theoretical model of YkoEDC complex. YkoE is shown in orange, the homology model of YkoC in gray, and the homology model of YkoD in aquamarine. (B) Theoretical model of YkoEC complex. YkoE is shown in orange, the homology model of YkoC colored according to conservation scores (cyan, variable; burgundy, conserved), the most conserved parts of YkoC are the helices CH2 and CH3, which are probably involved in interactions with YkoE and YkoD. An insertion and deletion in YkoC compared with EcfT could potentially result in some mechanistic differences between these T components. In YkoC, the loop connecting helix CH3 and transmembrane helix H5 is longer, and the hinge region between CH1 and CH2 is much shorter compared with the group II Ecf T component. The similarities in the pairwise alignments used to build the homology models are: YkoC and EcfT, 28%; YkoD N-terminal domain and EcfA2, 37%; YkoD C-terminal domain and EcfA1, 33% sequence identity.

None of the features characteristic for the group II transporters are present in YkoE, and this likely accounts for why YkoE does not associate with group II ECF modules (Figure 2C). Instead of the ΦxxxA motif, YkoE contains a semi-conserved S/AxxxI/VV motif located at the equivalent position on helix H1. This motif probably interacts with the CH2 helix of its T component YkoC. We modeled the YkoE-YkoC complex using a YkoC homology model and the EcfS(PanT)-EcfT complex as a template. The main interactions between YkoE and YkoC probably involve a large hydrophobic interface defined by helix H1 and the groove between H1 and H6 of YkoE and the highly conserved coupling helices CH2 and CH3 of YkoC. We speculate that the interaction between helix H1 and CH2 in the YkoE-YkoC complex is mediated by a hydrophobic/shape complementarity interaction between the branched amino acid I/V on the YkoE helix H1 and a highly conserved Gly144 on the YkoC helix CH2 (Figure 7). The lack of strong sequence conservation in helix H1 is probably due to the uniqueness of the YkoE-YkoC interface that was shaped during speciation through the co-evolution of the two binding partners.

Similar to the lack of a conserved motif on the helix H1, YkoE does not possess a deep groove that can accommodate the conserved Phe residues from the T-component helix CH3. The extended helix H2 in YkoE causes a displacement of the helix H6 that narrows the distance between helix H1 and H6 compared with other S components. In addition, the presence of two semi-conserved Phe residues (Phe19 and Phe26) on the helix H1 makes the groove between helices H1 and H6 very shallow (Figure S7B). Furthermore, helix CH3 in YkoC, which likely complements this groove in the YkoE-YkoC complex, contains highly conserved aliphatic residues in the equivalent positions of the Phe residues in the T component helix CH3.

Another difference between YkoE and group II S components resides in loop L1 (Figure 2). In YkoE, helices H1 and H2 are much longer than the corresponding helices in group II S components; the loops on the extracellular side joining adjacent α helices are short and seemingly do not serve any substrate-gating function. This is in stark contrast to the crystal structures of all S components determined to date, where loop L1 plays a major role in shielding the bound substrate molecule, usually resulting in very tight substrate binding (e.g. K_*d*_ of 100 pM in ThiT) (Figures 2 and S3) (Erkens et al., 2011, Zhao et al., 2015). Due to the absence of lid-like features in YkoE, it is likely that the conformational changes accompanying the substrate release in the context of the whole module differ from the group II S components and NikM, and probably involve a rearrangement of helices contributing to the substrate coordination.

Our structural analysis suggests that YkoEDC and group II ECF transporters have certain structural and mechanistic features in common. There are, however, pronounced differences between their components that could result in differences in their mechanism of vitamin transport. Further structural and functional studies of the YkoEDC complex would help to elucidate the key aspects of the mechanism of this transporter.

## Significance

**Energy coupling factor (ECF) transporters are essential vitamin transporters in many prokaryotes and are proposed to function via a unique toppling mechanism. We determined the structure of a thiamine-bound group I substrate-capture (S) component (YkoE), an integral membrane protein that is part of a dedicated tripartite transporter complex, in a lipidic environment. Our structure analysis revealed essential differences between YkoE and the better characterized group II ECF S components and uncovers how group I vitamin transporter S components can diverge from other group I and group II ECF transporters.**

## Experimental Procedures

### Cloning, Overexpression, and Purification of YkoE

The genes coding for *ykoE* were amplified from several bacterial species using PCR, cloned into a pNKE vector with an N-terminal His_6_ tag, and screened for expression using a variety of expression strains, media, and conditions. The most promising candidate, YkoE from *B. subtilis*, was overexpressed in *E. coli* Lemo21 cells (Schlegel et al., 2012) in terrific broth media supplemented with 1 mM L-rhamnose at 37°C. Cells were grown to an OD_600_ of 0.8–1.2, the temperature was lowered to 20°C, and 0.1 mM isopropyl thiogalactopyranoside was added. Cells were harvested the next day and lysed using an Avestin EmulsiFlex-C3 high-pressure homogenizer. Cell debris were pelleted at 20,000 × *g* for 30 min, and membrane fractions were isolated by centrifuging the supernatant at 200,000 × *g* for 1 hr. Membranes were resuspended and solubilized in buffer A (30 mM Tris-HCl, 500 mM NaCl, 5% glycerol, pH 7.1) with 1% n-dodecyl-β-D-maltopyranoside for 1 hr at 4°C; insoluble material was removed by centrifugation at 150,000 × *g* for 30 min. Solubilized membranes were incubated with Ni-NTA resin for 1 hr; the resin was washed with buffer A + 0.2% n-decyl-β-D-maltopyranoside and 50 mM imidazole. YkoE was then eluted with buffer B (30 mM Tris, 500 mM NaCl, 5% glycerol, 0.2% n-decyl-β-D-maltopyranoside, 250 mM imiadzole). Next, YkoE was incubated with TEV protease overnight to remove the His_6_ tag. TEV was subsequently removed by the re-application of the protein mixture to Ni-NTA resin. YkoE was further purified by size-exclusion chromatography using a Superdex S200 10/300 column equilibrated using buffer A + 0.2% n-decyl-β-D-maltopyranoside.

### Crystallization

Prior to crystallization, YkoE was concentrated to 10 mg/ml. Diffracting crystals could only be obtained using the LCP method with monoolein as lipid, despite extensive high-throughput screening of several homologs using the traditional sitting-drop vapor-diffusion method. Using LCP crystallization, several crystal hits were identified after several days at 20°C. Initial crystal hits exhibited poor diffraction, however addition of excess thiamine to the protein prior to crystallization and extensive crystal optimization improved the diffraction quality and resolution from 6.5 Å to below to 2 Å. The best crystals, diffracting to 1.95 Å, were obtained for protein purified in n-decyl-β-D-maltopyranoside and reservoir solutions containing 0.05 M disodium hydrogen phosphate, 19% PEG1000, 0.05 M citric acid, 0.1 M lithium sulfate, and 80 mM phosphoformic acid. SeMet-labeled YkoE was subjected to high-throughput screening after the crystals failed to grow in previously identified conditions. The best diffracting SeMet YkoE crystals were obtained in monoolein-based LCP at 20°C using reservoir solution containing 0.2 M ammonium phosphate monobasic, 0.1 M ammonium sulfate, 0.1 M sodium citrate (pH 4.5), and 32% PEG400. SeMet crystals of YkoE were much smaller in size (max. 20 μm) and diffracted to 3.2 Å. All crystals were flash frozen in liquid nitrogen without additional cryoprotection.

### Structure Determination

All X-ray diffraction data were collected at 100 K. Native data were collected at the PETRA III P13 and ESRF ID29 synchrotrons at 0.972 Å wavelength; data for SAD phasing on the SeMet-derivatized crystals were collected at the PETRA III P14 microfocus beamline at 0.979 Å wavelength. All datasets were processed with XDS (Kabsch, 2010) and merged with AIMLESS (Evans, 2006). Native YkoE crystals belonged to the I422 space group with cell dimensions a = 70.71 Å, b = 70.71 Å, c = 196.84 Å. SeMet-labeled YkoE crystals belonged to the C222_1_ space group with cell dimension a = 109.29 Å, b = 132.04 Å, c = 34.86 Å. SHELXD was used to find nine selenium atoms in the SAD dataset; SHELXE was used for initial density modification and a partial backbone building consisting of several α helices (Schneider and Sheldrick, 2002, Sheldrick, 2002, Sheldrick, 2008). Further rounds of density modification using RESOLVE allowed the placement of additional α helices in Coot (Terwilliger, 1999, Emsley and Cowtan, 2004). This model was then used for molecular replacement into a high-resolution native dataset. The final model was built using AUTOBUILD as well as manual building performed in Coot (Emsley and Cowtan, 2004, Terwilliger, 2003b, Terwilliger, 2003a). Refinement was carried out in *phenix.refine* (Afonine et al., 2012). Initial refinement steps included simulated annealing and optimization of atomic displacement parameters. At later stages, a thermal libation and screw-rotation (TLS) refinement strategy was used with the aid of TLSMD implemented as part of *phenix.refine* (Afonine et al., 2012). The final R factors of the refinement were 0.19/0.22 (R_work_/R_free_) with 99% of residues falling within the Ramachandran favored region and no outliers in disallowed regions, and a MolProbity (Chen et al., 2010) clashscore of 1.28.

### CD Spectroscopy

Far-UV CD measurements were made using a Jasco J-815 spectropolarimeter. Spectra were recorded from 260 to 190 nm using a 1 mm path length cell and 7–20 μM protein. CD melting curves were acquired by following the CD signal at 222 nm using a heating rate of 1°C/min. Buffer conditions were 30 mM Tris (pH 7.1), 200 mM NaCl, 0.2% n-decyl-β-D-maltopyranoside.

### Fluorescence Spectroscopy

Fluorescence measurements were performed on a Cary Eclipse fluorescence spectrophotometer. Intrinsic tryptophan fluorescence was measured with excitation at 280 nm and emission range from 300 nm to 500 nm. Individual spectra were measured using 200 nM YkoE protein in the absence and presence of 800 nM thiamine. Thiamine titrations were performed using 100 nM YkoE_M9_ and individual titrations of 2 μl. The excitation wavelength was 280 nm, and the emission signal at 340 nm and 350 nm was followed using an averaging time of 20 s. Buffer conditions were 30 mM Tris (pH 7.1), 200 mM NaCl, 0.2% n-decyl-β-D-maltopyranoside.

### Molecular Dynamics Simulations

The structures of the S components were processed by removing ligands and solvents. For FolT (4Z7F), PanT (4RFS), and RibU (3P5N), the missing loop was modeled using the Modeller interface implemented in Chimera (Yang et al., 2012), selecting the conformation with the lowest zDOPE score. The structures were converted to a CG model with the *martinize* program (de Jong et al., 2013). Subsequently, dipalmitoylphosphatidylcholine (DPPC) lipids and solvent were added in a cubic periodic box of 10 nm with the program *insane* (Wassenaar et al., 2015). The DPPC bilayer was built in the xy plane, with the protein TM helices at different angles to the z axis. Na^+^ and Cl^−^ were added at a concentration of 0.1 M to neutralize the system. The simulations were run with the GROMACS suite 4.6.5 (Hess et al., 2008, Pronk et al., 2013), using the MARTINI 2.2 force field (Marrink et al., 2007). The protein was simulated using the elastic network RubberBand, similar to ElNeDyn (Periole et al., 2009) and implemented in *martinize*. The systems were minimized with the steepest descent method during 500 steps using a time step of 20 fs. Next, equilibration runs were performed using a Berendsen barostat (Berendsen et al., 1984) with a coupling time of 10 ps. After the equilibration, production runs were done during 1 μs using semi-isotropic pressure coupling to a reference pressure of 1.0 bar with the Parrinello-Rahman barostat (Parrinello and Rahman, 1981) and a compressibility of 3.4 × 10^−4^ bar^−1^. The temperature was controlled at 323 K using the velocity rescaling thermostat (Bussi et al., 2007) with a time step of 1 ps. The final coordinates at 1 μs were transformed to GROMOS united atom representation (Oostenbrink et al., 2004) using the *backward* software (Wassenaar et al., 2014).

For the calculation of the protein toppling angle in the membrane, a vector was defined for each structure between CG backbone beads of two residues, spanning along the TM domain. The toggling angle was calculated between this vector and the z axis, using VMD (Humphrey et al., 1996).

**Table:** 

S Component	PDB	Vector
BioY	4DVE	153–182
FolT	4Z7F	7–131
HMP	4HZU	51–133
NikM	4M58	77–158
NikM_ΔC	4M5B	77–158
PanT	4RFS	162–192
RibU	3P5N	49–129
ThiT	3RLB	123–171
YkoE	5EDL	39–59
YkoE_ΔC		39–59

### Sequence Analysis/Bioinformatics

NCBI-NR was searched using PSI-BLAST (Altschul et al., 1997) to identify sequence homologs of YkoE, YkoC, and ThiT. Selected sequences were aligned, the alignment was manually corrected and then used as input for calculation of conservation scores. Multiple sequence alignments were generated using MUSCLE (Edgar, 2004) and Mafft (Katoh and Standley, 2013), and visualized using Jalview (Waterhouse et al., 2009). Evolutionary conservation was computed using ConSurf (Ashkenazy et al., 2010). Sequence logos were generated using WebLogo (Crooks et al., 2004). The substrate cavity was analyzed using DoGSiteScorer (Volkamer et al., 2012). HHpred (Hildebrand et al., 2009) was used to search the PDB and Structural Classification of Proteins databases for structural homologs. Homology models were generated with Modeller (Sali and Blundell, 1993) using EcfT, EcfA1, and EcfA2 structures as templates (PDB: 4RFS) and manually optimized alignments as input. Structure-based sequence alignment was produced manually using pairwise superpositions computed with TopMatch (Sippl and Wiederstein, 2008). Structure superpositions were made with TopMatch using a local constraint on the three N-terminal helices.

## Author Contributions

Conceptualization, I.J. and H.T.; Methodology, I.J., A.A., Y.A.H., and H.T.; Investigation, I.J., A.A., Y.A.H., and H.T.; Writing – Original Draft, I.J., Y.A.H. and H.T.; Writing – Review & Editing, I.J., A.A., Y.A.H., and H.T..; Funding Acquisition, H.T.; Supervision, H.T.

## Accession Numbers

Atomic coordinates and structure factors for the reported crystal structure have been deposited with the PDB under accession code PDB: 5EDL.

## Supplemental Information

Document S1. Figures S1–S7 and Table S1

Document S2. Article plus Supplemental Information
