# Supplementary material for: Crystal Structure of a Group I Energy Coupling Factor Vitamin Transporter S Component in Complex with Its Cognate Substrate
Source: Cell Chem Biol. 2016 Jul 21;23(7):827–36. doi: 10.1016/j.chembiol.2016.06.008 (PMC5037267; doi:10.1016/j.chembiol.2016.06.008)
Supplement: Document S1. Figures S1–S7 and Table S1 [file mmc1.pdf]

Cell Chemical Biology, Volume *23*

Supplemental Information

Crystal Structure of a Group I Energy Coupling Factor Vitamin Transporter S Component in Complex with Its Cognate Substrate

Inokentijs Josts, Yasser Almeida Hernandez, Antonina Andreeva, and Henning Tidow

Figure S1

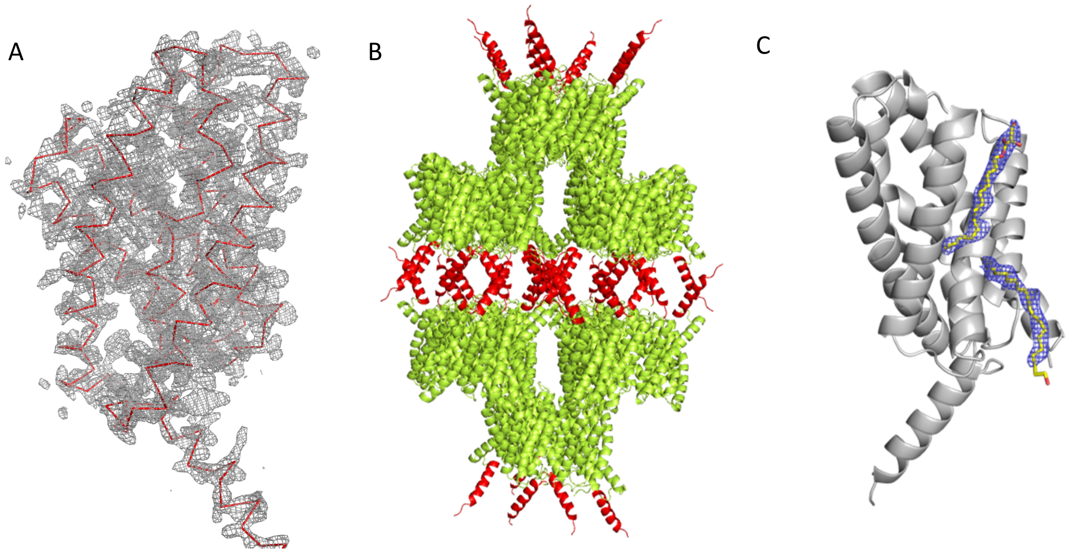

Crystallographic analysis of YkoE, related to Figure 1. A) Representative electron density of YkoE from the $2F_o$-F_c map contoured at 1.5σ. B) Crystal packing of YkoE. The packing is indicative of type I membrane protein crystals. YkoE molecules show extensive head-to-tail interactions mediated by the C-terminal helix H7 (red) and extracellular loops. C) Positions of the ordered monoolein molecules with their respective electron density from the $2F_o$-F_c map contoured at 0.7 σ.

Figure S2

A

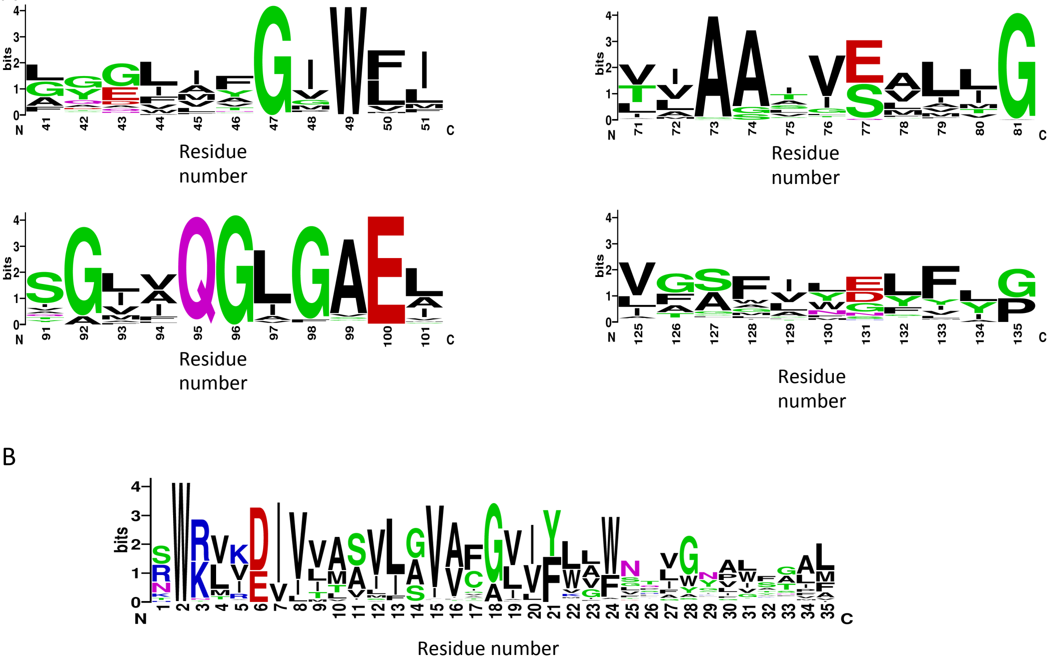

Conservation of thiamine coordinating residues in YkoE, related to Figure 4. A) Sequence logos illustrating the conservation of thiamine coordinating residues Y46, W49, E77, Q95, and D131. The residues coordinating the pyrimidine moiety (W49, E77, Q95) show particularly strong conservation. B) Sequence logo illustrating the semi-conserved S/AxxxI/VV motif located on helix H1.

Figure S3

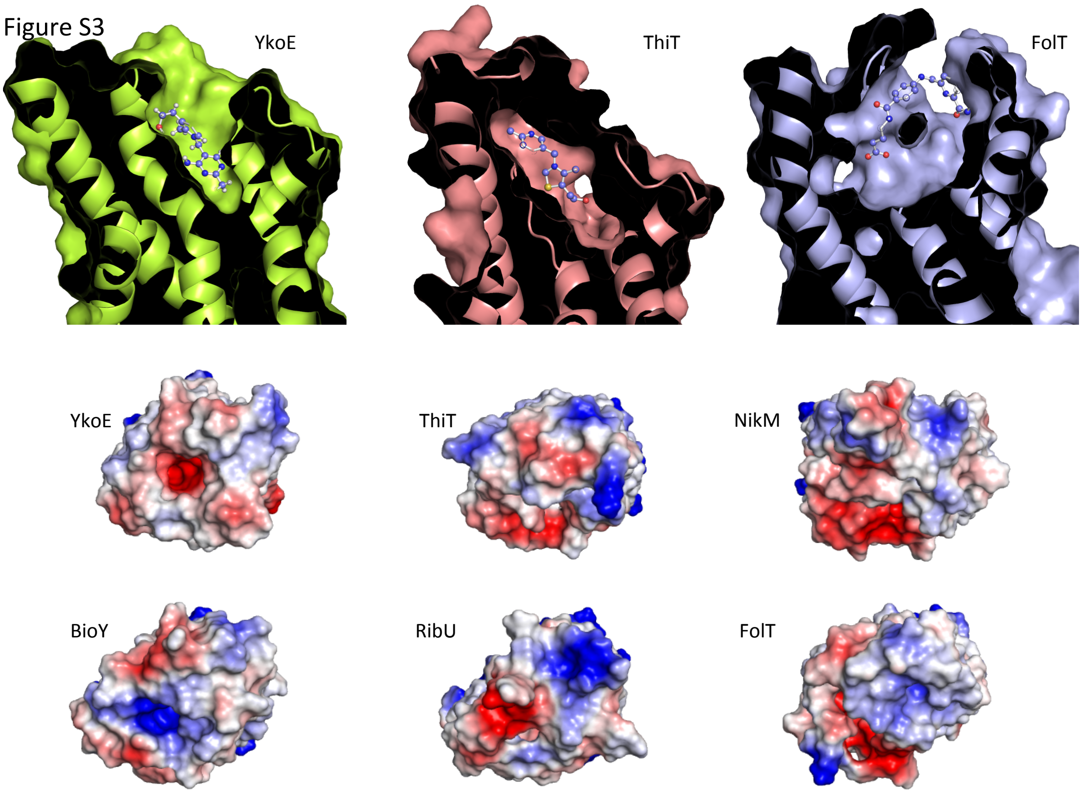

Different mechanisms for substrate entrapment between the group II S-components and YkoE, related to Figure 5. A) Cross-section of the substrate binding pockets between YkoE, ThiT and FolT highlights different mechanisms of substrate gating between the proteins. Both ThiT (light red) and FolT (light purple) utilize the extracellular loops (predominantly loop L1) in order to obstruct the substrate molecule within the cavity. YkoE (light green), on the other hand, does not possess any extensive extracellular loops that could act as a lid to lock the substrate and prevent the molecule from diffusing out. Thiamine is shown in grey sticks, with carbon atoms in purple, nitrogen in blue, oxygen in red and sulfur in yellow. B) Surface electrostatics of various S-components viewed from the extracellular side. All structures have been superimposed. Only YkoE shows an open binding pocket.

Figure S4

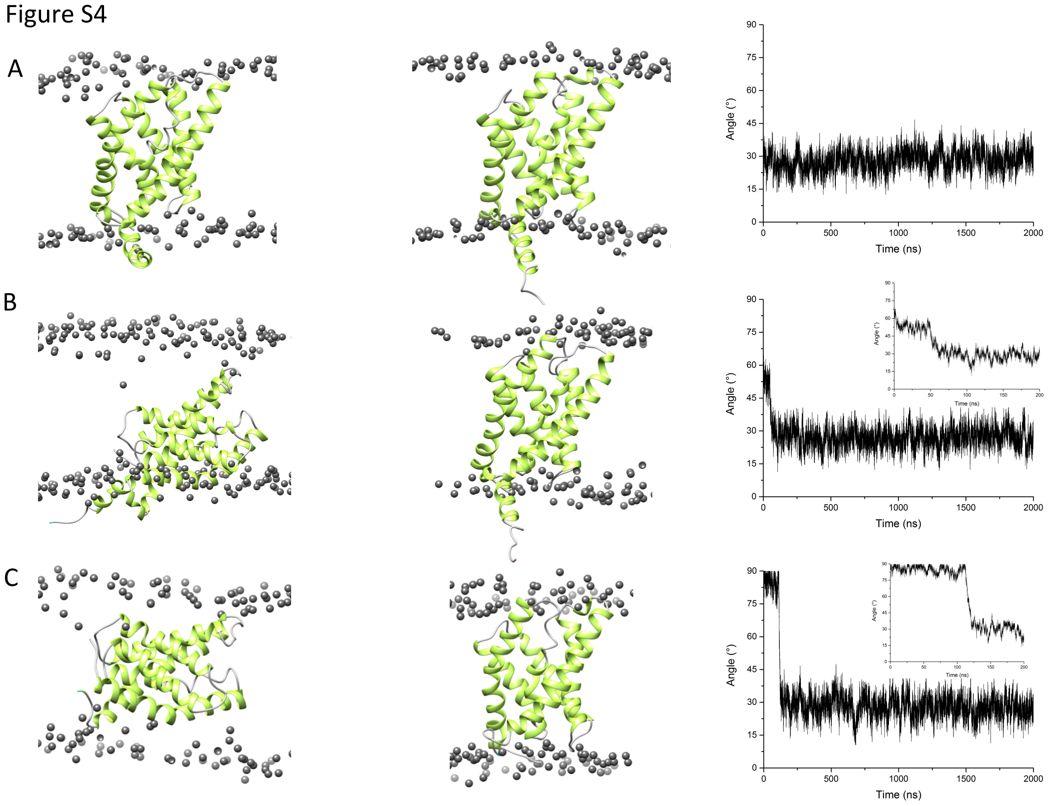

Coarse-grained molecular dynamics (CGMD) simulations to elucidate the orientation of YkoE in the membrane, related to Figure 7. (A and B) full-length YkoE, (C) ΔC-term YkoE. Left panel: starting orientation of YkoE in DPPC bilayer. Middle panel: Final orientation after a 2 μs CGMD simulation. Right panel: Toggling angle of the transmembrane domain in the bilayer during the simulation. The inset graphs in B and C represent a zoom of the first 200 ns.

Figure S5

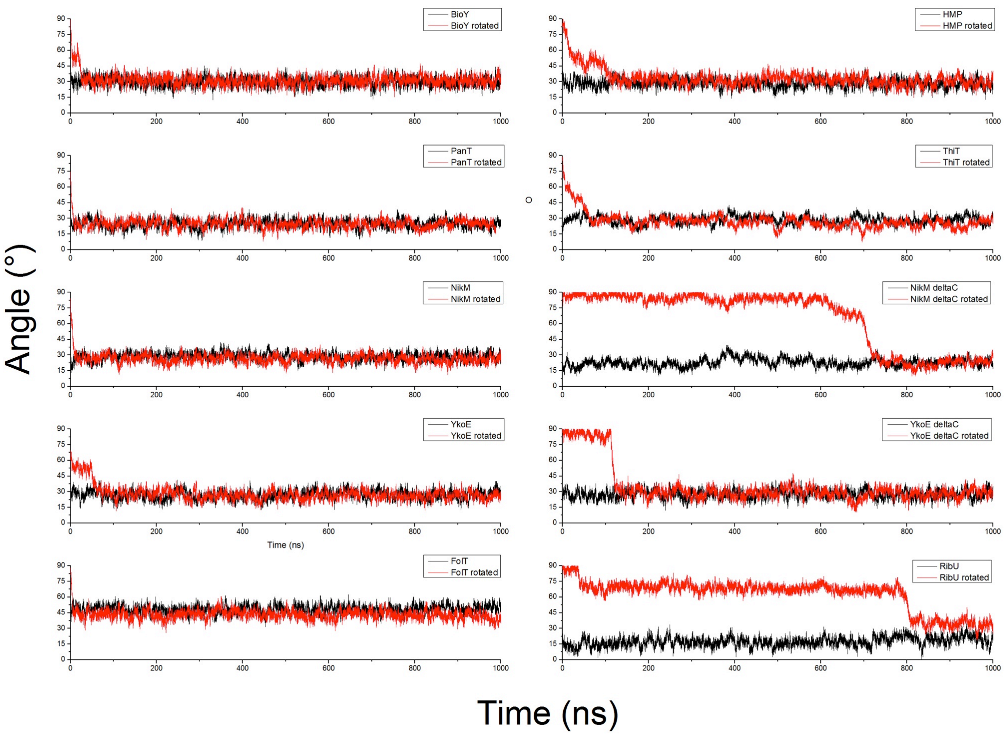

Coarse-grained molecular dynamics (CGMD) simulations to elucidate the orientation of various group II S-components in the membrane, related to Figure 7. The black trajectory represents a standard starting orientation; the red trajectory represents a parallel starting orientation.

Figure S6

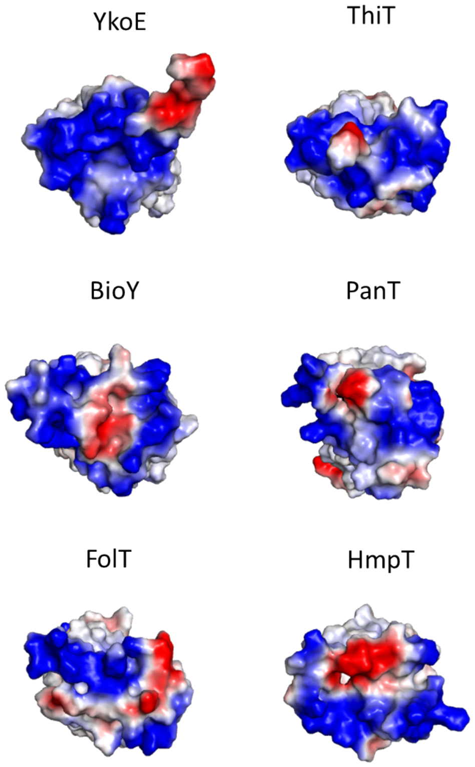

Surface electrostatics of various S-components viewed from the cytoplasmic side, related to Figures 2 and S5. The assignment of cytoplasmic side is based on the positive inside rule and physiological requirements. All structures have been superimposed.

Figure S7

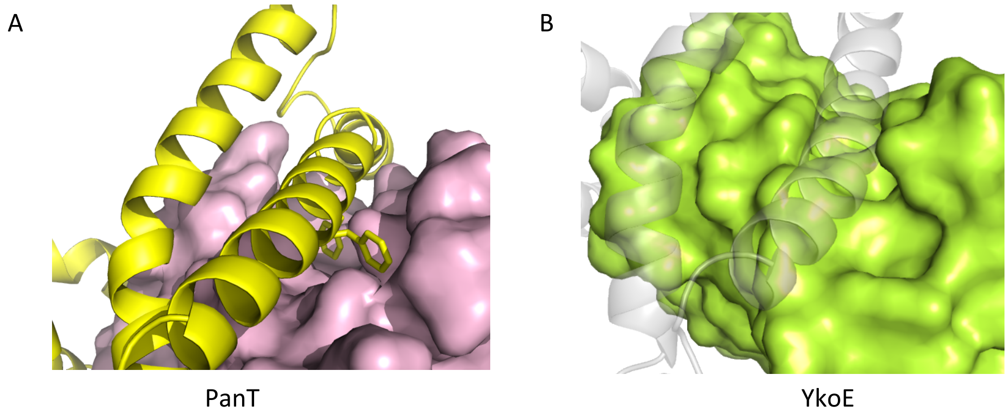

Comparision of T-component binding grooves between YkoE and PanT in complex with the ECF module, related to Figure 7. All the group II S-components studied to date contain a surface groove between helix H1 and helix H6, which accommodates the coupling helices from the T-component containing two conserved Phe residues. A) This groove is evident in PanT (light pink) with EcfT coupling helices shown in yellow. B) In YkoE (light green) this groove is significantly shallower due to helix rearrangements and the presence of two Phe residues in helix H1. Helix CH3 in YkoC, that likely complements this groove in the YkoE-YkoC complex, contains highly conserved aliphatic residues in the equivalent positions of the Phe residues in the T-component helix CH3. The coupling helices of YkoC (modelled on PDB: 4rfs) are shown in light grey.

Table 1 Data collection and refinement statistics (molecular replacement), related to experimental procedures

	Native YkoE	SeMet YkoE
**Data collection**		
Space group	I 4 2 2	C 2 2 2_1
Cell dimensions		
a, b, c (Å)	70.71, 70.71, 196.84	109.3, 132, 34.96
α, β, γ (°)	90, 90, 90	90, 90, 90
Resolution (Å)	44.58-1.95(2.02-1.95)	84.2-3.2(3.42-3.20)
R_{merge}	0.096(0.727)	0.19(1.224)
$I / \sigma I$	11.23(2.30)	10(2.3)
Completeness (%)	99.94(100)	100(99.9)
Redundancy	7.0(7.0)	12.4(12.8)
**Refinement**		
Resolution (Å)	1.95	
No. reflections	18753 (1855)	
$R_{\mathrm{work}} / R_{\mathrm{free}}$	0.20/0.22	
No. atoms	1607	
Protein	1476	
Ligand/ion	57	
Water	74	
B-factors		
Protein	33.54	
Ligand/ion	49.20	
Water	42.50	
R.m.s. deviations		
Bond lengths (Å)	0.007	
Bond angles (°)	0.92	

*Values in parentheses are for highest-resolution shell.
